# Surface plasmon resonance based on molecularly imprinted nanoparticles for the picomolar detection of the iron regulating hormone Hepcidin-25

**DOI:** 10.1186/s12951-015-0115-3

**Published:** 2015-08-27

**Authors:** Lucia Cenci, Erika Andreetto, Ambra Vestri, Michele Bovi, Mario Barozzi, Erica Iacob, Mirko Busato, Annalisa Castagna, Domenico Girelli, Alessandra Maria Bossi

**Affiliations:** Department of Biotechnology, University of Verona, Strada Le Grazie 15, 37134 Verona, Italy; Center for Materials and Microsystems CMM-MNF, FBK Fondazione Bruno Kessler, Via Sommarive 18, 38123 Povo-Trento, Italy; Department of Medicine, University of Verona, Section of Internal Medicine B, 37134 Verona, Italy

**Keywords:** Molecularly imprinted polymers, Nanoparticles, Hepcidin, Biosensor, Surface plasmon resonance, Iron metabolism

## Abstract

**Background:**

Molecularly imprinted polymer (MIP) technique is a powerful mean to produce tailor made synthetic recognition sites. Here precipitation polymerization was exploited to produce a library of MIP nanoparticles (NPs) targeting the N terminus of the hormone Hepcidin-25, whose serum levels correlate with iron dis-metabolisms and doping. Biotinylated MIP NPs were immobilized to NeutrAvidin™ SPR sensor chip. The response of the MIP NP sensor to Hepcidin-25 was studied.

**Findings:**

Morphological analysis showed MIP NPs of 20–50 nm; MIP NP exhibited high affinity and selectivity for the target analyte: low nanomolar Kds for the interaction NP/Hepcidin-25, but none for the NP/non regulative Hepcidin-20. The MIP NP were integrated as recognition element in SPR allowing the detection of Hepcidin-25 in 3 min. Linearity was observed with the logarithm of Hepcidin-25 concentration in the range 7.2–720 pM. LOD was 5 pM. The response for Hepcidin-20 was limited. Hepcidin-25 determination in real serum samples spiked with known analyte concentrations was also attempted.

**Conclusion:**

The integration of MIP NP to SPR allowed the determination of Hepcidin-25 at picomolar concentrations in short times outperforming the actual state of art. Optimization is still needed for real sample measurements in view of future clinical applications.

**Electronic supplementary material:**

The online version of this article (doi:10.1186/s12951-015-0115-3) contains supplementary material, which is available to authorized users.

## Background

The analytical determination of a target molecule is dependent on its specific interaction with a recognition element [1, 2]. Thus in sensing technologies, both knowledge on the intermolecular interactions and the ability to control over the recognition processes are keys to improve and enhance the sensitivity and the selectivity of the system [3, 4]. Aiming at increasing the sensor performance significant efforts are put on the improvement of the recognition element. Biological recognition, principally actuated by antibodies and receptors, is known to offer exquisite specificity and selectivity, while often lacking of long term stability, since proteinaceous material exhibits the tendency to unfold when coupled to the transducer. Thus synthetic recognition elements are gaining increasing importance in the field [3, 5]. Among the methods for their preparation, the molecular imprinting of polymers (MIP) is a versatile technology for imparting entailed recognition properties to polymeric materials [6–8]. The concept was established in 1972 by Wulff and Sarhan, when they investigated a new method to introduce functional groups with specified stereochemical properties into polymers [9]. Briefly MIPs are produced via a template assisted synthesis: (1) the target analyte, called the template, is solvated together with selected functional monomers, these latter re-organize around the template in a thermodynamically driven process that lead to the minimum configuration energy and to the formation of template-monomers pre-polymerization complexes (2) with the addition of the crosslinker and the initiators the material is polymerized, (3) at the end of the synthesis, unreacted monomers and the template are removed by washings, leaving a polymer with molecular cavities complementary to the template exposed and prone for rebinding.

Inherent advantages of MIPs are the stability of the polymeric material to harsh conditions, the cheap synthesis, the processability, the ease of integration to sensors and the great flexibility of the imprinting strategy, which allows in principle to prepare binding materials for whatsoever target analyte (from small molecules to proteins, to bacteria and viruses and to impart the desired degree of affinity [10, 11]. MIP selectivities and affinities have been reported on the par of natural antibodies [12, 13]. For all these reasons the exploitation of MIPs as mimic of the biological recognition element is well consolidated in analytical technologies [i.e. chromatography, capillary electrophoresis (CE)] [14], assays [12] and sensors [10, 15–17]. So far, the majority of the reported MIPs have been imprinted in organics and the majority of the targets are small analytes, while the current state of the art addresses the issues of imprinting complex molecules aqueous solvated, such as proteins, hormones and signal peptides, for whose water compatible MIPs are under development [18–20]. A further frontier research area focus on the structural architecture of the MIP polymer which is rapidly evolving from the more traditional macropolymeric bulk material, to micro and nanostructures [21]. Undoubtedly, MIP macrostructures have been offering high loading capacity which is of advantage for their incorporation into a sensor. At the same time the physical limitations inherent with the macrodimensions often brought the sensing process to only partial success. Specific limitations associated with the macrodimensions are the inhomogeneity of the macroparticulate, which results in lack of uniformity of the polymer fragments and non consistency of the quantity of available binding sites per polymer macroparticle; these affect both the time of response of the sensor (i.e. the equilibrium time is not constant; the signal stabilization could require hours) and the reproducibility of the response. Moreover the MIP macroparticulate exhibited scarce compatibility with some transduction forms, such as surface plasmon resonance (SPR) detection. In SPR the binding event shall occur within a depth of 200 nm from the metal surface, i.e. a distance within which the analyte-receptor interaction effectively perturbs the plasmonic wave and generates a detectable signal, yet such distance is rather incompatible with macrostructured MIPs [22].

Nowadays nanometric sized objects have been demonstrated to posses unique molecular, physical and chemical properties [23]: increased surface to volume ratio, less defects in the material, i.e. monodispersity of the nanoparticle (NP) population, homogeneity of the binding sites, equivalent number of binding sites per NP, higher accessibility of the binding sites, distinctive optical properties, i.e. discrete light absorbtion or emission, badgap etc. [23] are among the key features of nanomaterials, which have been allowing the development of a new generation of sensors with superior performances respect to the former macro-material-based [3, 4, 24]. In case of MIPs, nanoMIPs have been outperforming macroMIPs sensitivity and detection limits of at least three orders of magnitude [22, 25, 26], thus nanometric sized MIPs are being pursued. Protocols for downsizing MIPs to NPs or nanofilms are foreseen as strategic for the success of MIP-sensing [9] with important impact on real time monitoring [11].

Nano-MIPs are reported in the form of nanosurfaces, i.e. thin films, or NPs [9, 27, 28]. These can be produced either by blending MIPs into supporting or sacrificial nanostructured materials [29], or by core–shell synthesis over polymeric or inorganic nanostructures, such as quantum dots, silica or metallic NPs [30, 31], or by synthesizing whole polymeric MIP NPs [32, 33]. In the present account, entirely polymeric MIP NPs, prepared through a precipitation polymerization protocol [32], were studied. These were selected for their favorable characteristics of high yield, water compatibility, small dimensions (10–100 nm), which suit well our goals to target a peptide biomarker and to be integrated into a SPR sensor in similarity with antibodies.

Target analyte for this study was the peptide hormone Hepcidin-25 which plays a key role in iron homeostasis, being the only regulator of the iron efflux from storage cells, i.e. macrophages and hepatocytes, to serum [34–37]. The mature form of the hormone, Hepcidin-25, is a biomarker for iron dis-metabolisms, inflammations and doping [27, 38]; its determination in serum helps in the clinical assessment of the different iron metabolism disorders, offering indications for the prognosis and therapeutic interventions [39–44]. Moreover, the tight link between iron metabolism and erythropoietic activity correlates Hepcidin-25 levels to blood doping in athletes [38]. Whereas the circulating truncated forms of the hormone, i.e. Hepcidin-24, 22 and 20, progressively lacking of portions of the N terminus, appear not to be involved in the iron regulation and still have unknown roles [45–47].

Despite the interests for the quantitative determination of Hepcidin-25, the structural characteristics of this hormone hampered its detection for quite a long time. In particular, Hepcidin-25 has a rigid structure kept in place by four disulphide bridges [48, 49], small dimensions (2789 Da) and a high degree of conservation through the evolutive scale, characteristics that hampered the production of antibodies up to very recent years [50, 51]. Methods based on mass spectrometry (MS) were proposed as an alternative [39, 52–54]. Nevertheless the quantitative determination of Hepcidin-25 is still an open issue, as resulted by round-robin studies that compared the actual hepcidin quantification methods [55].

**Fig. 1:**
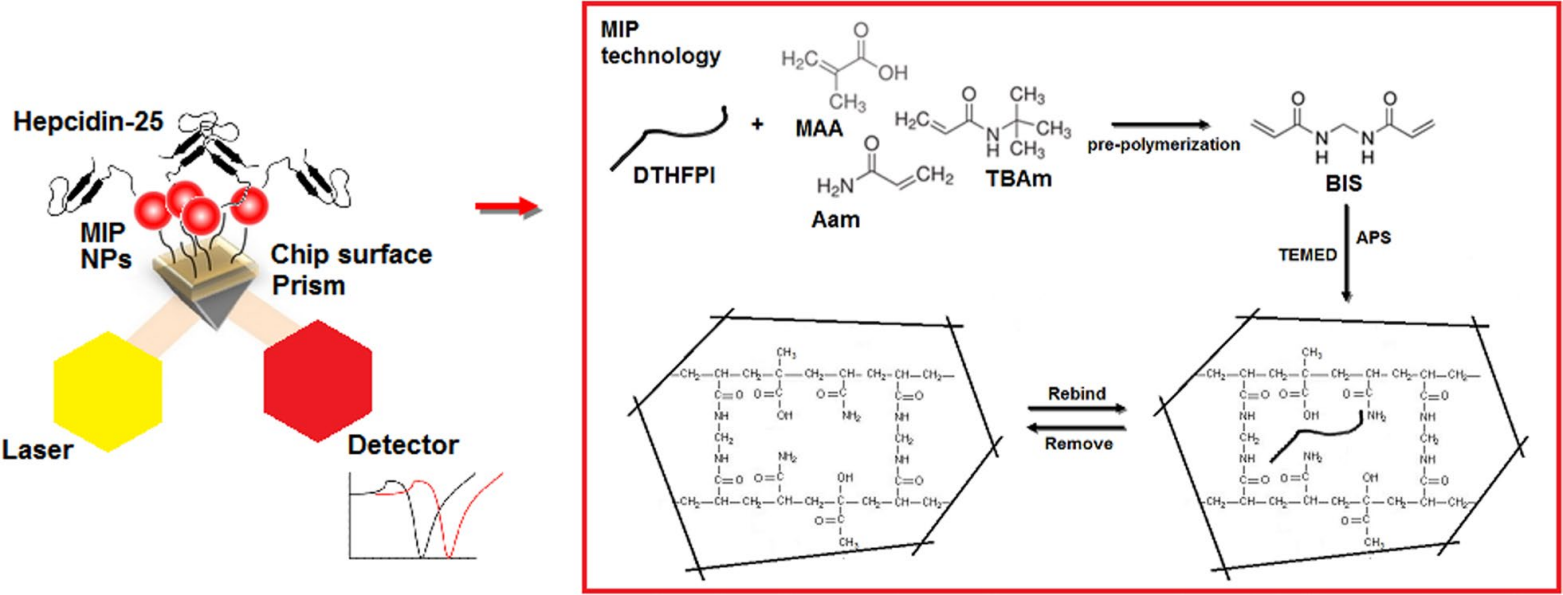
Scheme of MIP NP SPR sensor. The MIP NPs anchored onto the chip surface through the biotin-NeutrAvidin™ bond are represented as *red dots*, the *inset* shows the MIP synthesis

The MIP strategy for Hepcidin-25 determination was also attempted in 2010 [56]. In the case, MIP hydrogels of about 100 micrometer in size were prepared in aqueous solution and targeted the N terminus peptide of Hepcidin. Results indicated that the microMIP had an imprinting factor (IFs) of about 1.1–1.5 with an incubation times of about 24 h [56]. As a consequence the microMIP material was rather inconvenient both for sensing and for clinical assays. In the present study, we aimed at optimizing the MIP composition for the recognition of Hepcidin-25, to miniaturize the MIP to nanomaterial for its further integration into an optical sensor for the real time monitoring of the hormone. We thus prove the possibility to imprint polymeric NPs with the N-terminus portion of the hormone, with the goal to produce easy to synthesize and selective recognition elements able to discriminate the bioactive full length Hepcidin-25 from truncated hepcidin. As shown in Fig. 1, the MIP NPs were integrated into optical SPR sensor and the performance of the sensor was evaluated in model solutions, proving the possibility to detect the hormone at picomolar levels.

## Results and discussion

### Rational design, synthesis and physical characterization of MIP NPs

The selection of the template was based on the epitope imprinting strategy [57–59]. The N-terminus hexapeptide of Hepcidin-25, with sequence DTHFPI, was therefore designated as template both for its putative role in triggering the iron-regulation response [60], and for the short and linear sequence, which can be imprinted with higher fidelity respect to the full hormone, that retain a hairpin secondary structure [57]. The same template selection was adopted earlier by Abbate [56]. Considering the peptidic nature of the template and its amphipathic character, we aimed at preparing an aqueous compatible polymer composed of a variety of monomers, i.e. neutral, hydrophobic and charged, in order to match all the different functionalities of the aminoacids with complementary weak interactions: hydrogen bonds, hydrophobic, electrostatic. This was achieved with the combination of: acrylamide (Aam), tert-butylacrylamide (TBAm), diethyl aminoethyl methacrylate (DEAEm) and methacrylic acid (MAA) monomers, as successfully indicated by Hoshino [32]. Bisacrylamide was the crosslinker. Differently from the work published by Shea’s group [32], we decided to use the 80 % w/v of crosslinker, to confer and maintain a certain degree of rigidity in the polymer, in fact in previous attempts with lowly crosslinked macrostructures (<5 % C) we encountered the problem of over-rehydration of the hydrogel [61], which at a molecular level results in a deformation of the recognition cavities or in less accessibility of the binding sites. Moreover the crosslinking level was similar to previous submicron sized hydrogels, mimic of biomolecules inhibitors, that have been reported to have exquisite interaction with the active site of enzymes [33].

The MIP selection of the charged monomers was done with the aid of molecular modelling [13]. Here, the N terminus of Hepcidin-25 was docked with a combination of charged (either DEAEm or MAA) and neutral monomers (Aam): to mimic the pre-polymerization solution two monomers of a same charge and two neutral monomers were funneled through the HADDOCK software. At the end of the simulation the HADDOCK score values, that indicate the strength of the complex, showed a stronger interaction for MAA/template (−68.0 a.u.) respect to DEAEM/template (−59.2 a.u.), thus MAA was used for the syntheses (details in Additional file 1).

The synthesis of the NPs was conducted with a total monomer concentrations of 14 or 35 mM, in water supplemented with 0.02 %, SDS [32] and triggered with the chemical free-radical-generating pair TEMED/APS, for 17 h in order to let the process reach the completion [32]. Respectively for 14 and 35 mM total monomers, the initiator was the 3.5 % and the 7 % of the whole polymerizable double bonds in the solution. Such ratio, slightly higher than that used for the preparation of hydrogels (ca. 1.5 % initiator vs. mol of polymerizable double bonds) balanced the necessity to produce porous nanomaterial compatible with the a fast equilibration of the peptide Hepcidin with the genesis of many nucleation centers elongating for shorter lengths, which should result in NPs <100 nm.

Different template concentrations (32 and 200 µM) were tested in the synthesis in order to assess the influence of the template vs. functional monomers ratio (mol:mol) over the MIP NP recognition properties (polymeric compositions in Additional file 2). Control, non imprinted, polymers, NIP NPs, were prepared in an identical manner but in the absence of template. The calculated yield for the synthesis was >90 %.

**Fig. 2:**
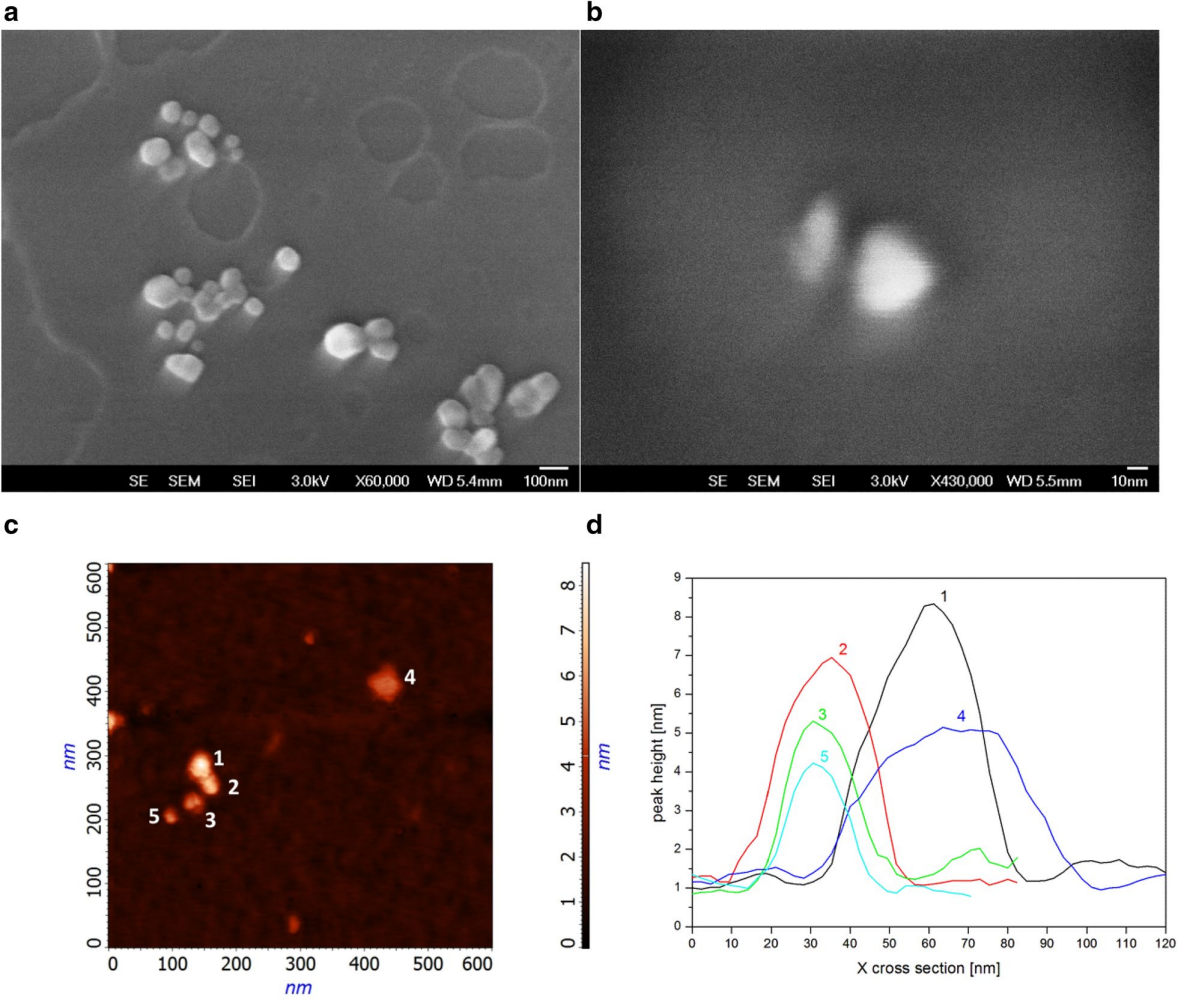
Morphological analysis of the MIP NPs. SEM (**a**, **b**) and AFM (**c**, **d**) analysis gave morphological information on the MIP NPs. SEM images indicated a mean size of 55 ± 7 nm. The AFM measured a mean size of 27.4 ± 8.7 nm for the nanoparticles (**d** the length distribution in nm of five NPs was: #1 34.3; #2 25.2; #3 22.6; #3 20.5; #4 41.7; #5 20.1). The MIP NPs deposition indicates the presence of both single nanoparticle (ca. 22 nm) and small aggregates of dimensions <100 nm. The data are in agreement with the results of the DLS analysis

The physical characterization of the NPs was performed with Dynamic Light Scattering (DLS), Atomic Force Microscopy (AFM) and Scanning Electron Microscopy (SEM). DLS data are reported in Table 1, while the morphological information of the NPs are shown in Fig. 2.

**Table 1:** Physical properties of the NPs

Total monomer concentration (%)	Template concentration (µM)	Size (nm)	Mn (kDa)	PDI	Zeta potential (mV)
0.2	–	29 ± 0.3	1000	0.3	−10 ± 1
	32	36 ± 1	1100	0.4	−12 ± 1
	200	26 ± 0.1	700	0.3	−10 ± 1
0.5	–	55 ± 0.1	2600	0.2	−15 ± 1
	32	52 ± 2	3500	0.2	−12 ± 1
	200	69 ± 1	3400	0.2	−13 ± 1

Table 1 reports the physical properties for each NP composition polymerized (NIP, MIP with 32 µM template, MIP with 200 µM template, at 0.2 % T and at 0.5 % T). In general, the low polydispersity indexes measured (PDIs <0.3), indicated that uniform populations of NPs were synthesized by the precipitation polymerization method. As an exception, the MIP recipe 0.2 % T-32 µM template resulted in a higher inhomogeneity of the nanomaterial (PDI 0.4).

The size distributions of the NPs ranged from 26 ± 0.1 to 36 ± 0.3 nm for the syntheses conducted at 0.2 % T, while dimensions between 55 ± 0.1 and 69 ± 1 nm were observed for the syntheses conducted at 0.5 % T (see Table 1). A positive correlation between the particle dimension and the quantity of monomers in the synthesis protocol (% T) was observed. The same trend was seen for the estimated mean molecular weights (Mn) and % T: the 0.2 % T NPs were of about 700–1000 kDa, whereas the 0.5 % T NPs were of about 3500 kDa.

The dimension of the NPs was independently evaluated by electron microscopy measurements (SEM Fig. 2a, b; AFM, Fig. 2c, d): the average diameters of the NPs resulted of ca. 27.4 ± 8.7 nm (Fig. 2d); qualitatively the NPs population appeared to include both single particles and small aggregates. DLS data were in agreement the electron microscopy measurements, despite the different rehydration state of the NPs in the two analyses could explain small differences (DLS measures rehydrated NPs and over-estimate the size, AFM and SEM measure in dry state where the NPs shrunk).

The differences reported for the NPs prepared at 0.2 and 0.5 % T (both MIP and NIP) were not substantial, thus to our judgement the best protocol was 0.5 % T, which ensured a higher quantity of NPs production per synthesis batch. Overall the results indicated that the NP characteristics were comparable to those of large protein complexes. This is reported to give advantages in terms of fast binding equilibria, compatibility with fluidic system (no clogging of tubings) [24], compatibility with protein handling protocols and limited number of binding sites per particle [62]. Therefore the synthesized MIP-NPs were compatible in dimension with the final goal of sensor integration.

### Investigation over the MIP NPs recognition properties

The MIP NPs were then tested for their recognition properties towards their putative ligands by the isothermal titration calorimetry (ITC) technique. The ITC method measures the heat exchanges associated with the formation of a complex, offering thermodynamic insights into the analyte/NP association. Here 1.2 µM of NP (MIP or NIP, called titrand) were titrated in 12 sequential injections of a 4 µM solution of injectant (i.e. the hexapeptide template, or Hepcidin-25, or Hepcidin-20, or a non related peptide of 10 aminoacids (NR10), or a scramble sequence of the template). The heat generated upon each injection was recorded, integrated and corrected by the subtraction of the heat of dilution contribution (i.e. injectant titrated into pure buffer). Corrected heat areas expressed in kJ/mol were plotted versus the molar ratio titrand/injectant and fitted with a single point equation (see Additional file 3 for details).

The resulting thermodynamic data are reported in Table 2. All the binding events exhibited a negative ΔG°. The library of MIP NPs did bind the template hexapeptide with very low nanomolar dissociation constants (3–7 nM), indicating the high affinity of the MIP NP/template interaction. The stoichiometry of the complex (n, Table 2) showed an interaction of about 1 mol of peptide and 0.5 mol of MIP NP, result that, given the polydispersity of the NPs and the possibility to form aggregates in solution, can suggest a low number of high affinity binding sites per MIP NP, in analogy with other MIP NPs [62, 63]. An estimation of the binding capacity of the MIP NPs was attempted from the parameter n and resulted in ca. 2 µg/mg of MIP NPs.

**Table 2:** Thermodynamic constants for the binding of MIP NPs to different analytes measured by Isothermal Titration Calorimetry (ITC)

NP	Ligand	Kd (nM)	n	ΔH (kJ/mol)	ΔG (kJ/mol)
02 MIP32	DTHFPI	6 ± 3	0.4 ± 0.1	−261 ± 18	−47 ± 2
02 MIP200	DTHFPI	7 ± 1	0.4 ± 0.1	−128 ± 25	−46 ± 1
05 MIP32	DTHFPI	3 ± 1	0.4 ± 0.1	−177 ± 14	−49 ± 1
05 MIP200	DTHFPI	5 ± 3	0.5 ± 0.1	−131 ± 4	−48 ± 2
05 MIP200	Hepcidin-25	13 ± 2	0.3 ± 0.1	−463 ± 46	−51 ± 9
05 MIP200	Hepcidin-20	–	–	–	–
05 MIP200	THFDPI	19 ± 5	0.5 ± 0.1	−395 ± 27	−37 ± 2
05 MIP200	NR10	–	–	–	–
02 NIP	DTHFPI	–	–	–	–
05 NIP	DTHFPI	–	–	–	–
05 NIP	Hepcidin-25	–	–	–	–

Experiments were performed by titrating 1.2 µM NPs with 4 µM ligand: dissociation constant (Kd), binding stoichiometry (n), enthalpy and Gibbs free energy variation. The heat contribution of control NPs was subtracted from MIP NPs, thus the resulting interactions are primarily driven by specific binding sites. Thermograms in Additional file 1

The MIP NPs did bind full length Hepcidin-25 with a Kd of 13 nM. The affinity, the stoichiometry and the exothermic effect for the binding of MIP NPs/Hepcidin-25 were comparable to that of the binding MIP NP/hexapeptide and were indicative of the successful recognition of the template by the imprinting cavities, also when the N terminus hexapeptide is part of a structured polypeptide, such as the folded Hepcidin-25. These data corroborate and support the validity of the epitope strategy [57–59]. Moreover no binding was observed when MIP NPs were challenged with the truncated Hepcidin-20, lacking of the N terminus. This accounted for the selectivity of the MIP NPs and fully supported our initial hypothesis of targeting the N terminus of the hormone in view of developing an analytical system for the discrimination of the iron regulating form.

The selectivity of the MIP NPs was further confirmed in a titration experiment with a peptide having an unrelated peptide sequence (i.e. NR10): no interaction was measured. On the contrary, when the scramble hexapeptide sequence was tested for its recognition by the MIP NPs a high affinity was measured (despite a slightly less favourable energy was implicated). A possible explanation implies that the recognition mechanism is mainly driven by the steric hindrance and by the surface charge density of the peptide, instead of relying on a strict sequence specificity.

All the controls performed titrating the NIP NPs with the template indicated no measurable binding. This was indeed another evidence of the efficacy of the imprinting process to generate specific binding sites. Thus, we investigated further the genesis of the recognition sites on the NPs during the imprinting process, setting up an experiment in which the template was titrated to each MIP NPs of our library, i.e. made with: (1) different quantities of template (32 or 200 µM) and (2) different total monomers concentration during the polymerization batches (0.2 and 0.5 % T), which corresponded respectively to the following template:monomer molar ratio 1:88; 1:222; 1:14; 1:35. As shown in Table 2, no significant changes or trends were evidenced by the thermodynamic parameters within the NP library. It could be hypothesized that the formation of the imprinted cavities during the precipitation polymerization is not merely controlled by the quantity of monomers vs. template present in the polymerization solution. This raise new questions for the imprinting community about the rationalization and control over the MIP NPs synthesis. Few observations on the role exerted by the monomer composition in the recognition have been proposed in the recent past [64–66] and might fit to answer the here proposed question.

To summarize the functional data: overall the MIP NPs exhibited low nanomolar dissociation constants for their template and for Hepcidin-25, indicating exquisite recognition abilities on the par of monoclonal antibodies, and attuned selectivities that allows the discrimination of the active form of the hormone. These characteristics were considered appropriate for exploiting the behaviour of our MIP NPs in sensing. Moreover the MIP NPs lyophilized and stored at 4 °C did retain their binding properties form >6 months, confirming the stability of these synthetic recognition elements and accounting for their potential impact in substituting biomolecules in sensors.

### Preparation of the MIP NP SPR sensor

The MIP NPs have been used earlier as synthetic recognition element in a surface plasmon resonance (SPR) microfluidic sensor [9, 67]. Here, the chosen surface suitable for the MIP NPs immobilization was the commercially available gold chip coated with NeutrAvidin™ (BioCap). At first the MIP NPs were derivatized with biotin-PEG-amine in order to introduce both the binding functionality and a spacer arm of 8 residues to prevent the steric hindrance of the particle at the chip surface (derivatization protocol and control experiments in Additional file 4). The size distribution of control and biotinylated NPs was determined by DLS: MIP NPs showed a hydrodynamic diameter of 52 nm (±2) and a PDI of 0.2, while biotinylated MIP NPs had a diameter of 139 nm (±2) with a PDI of 0.3 (Additional file 4: Figures 4.1 and 4.2).

**Fig. 3:**
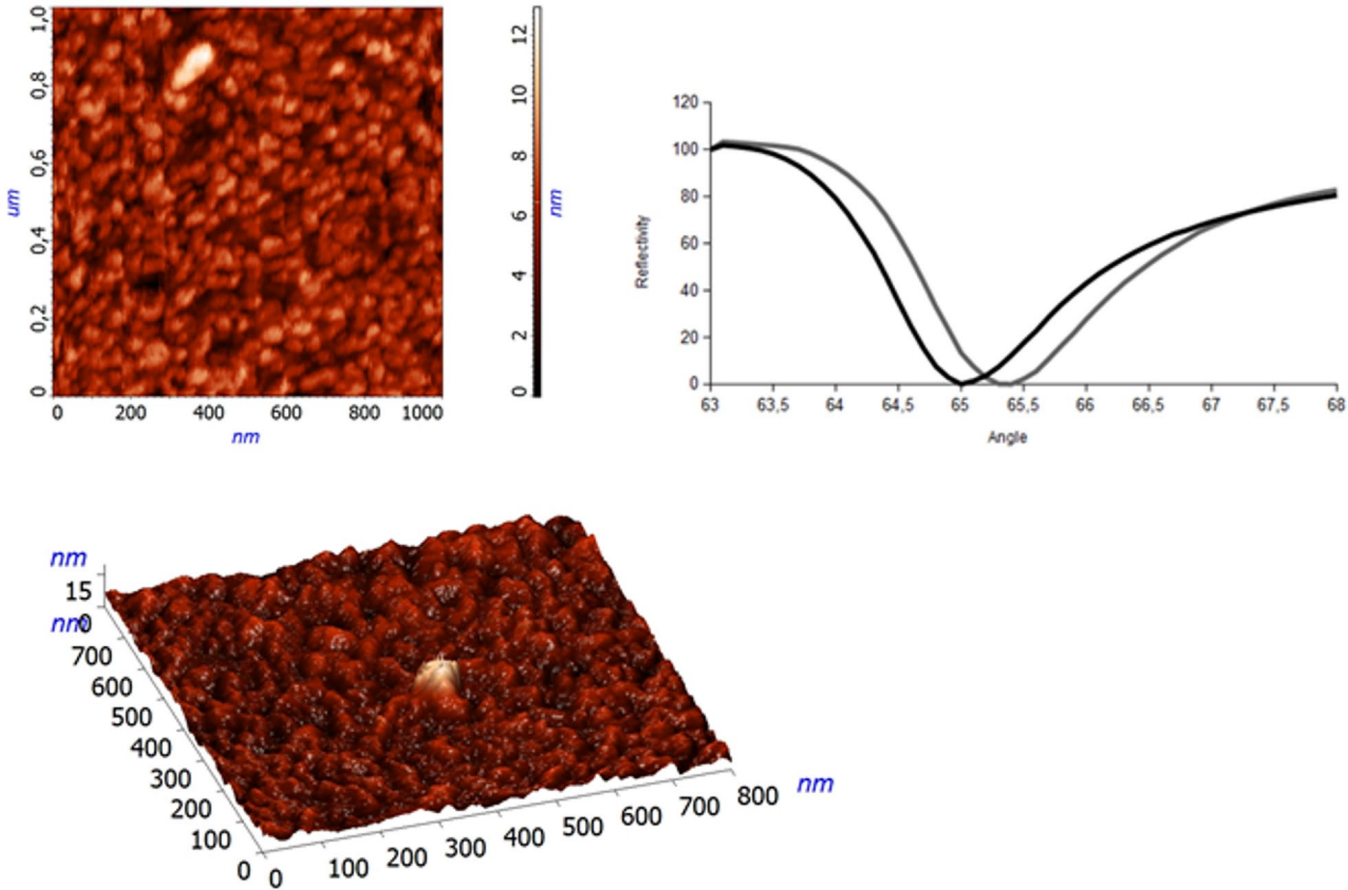
Morphology of the chip sensor surface modified with MIP NPs. The morphology of the chip after MIP NP derivatization was investigated with AFM. The surface appeared uniformly covered with MIP NPs. The *dips panel* shows the SPR minima of one of the three channels before (*black signal*) and after (*grey signal*) the derivatization of the chip surface with MIP NPs. Each dip shifts to the *right* (higher SPR angles) when the mass increases on the corresponding sensing surface

Then the MIP NPs were dispersed in HBS to a final concentration of 1 mg/ml and bound off line to the NeutrAvidin™ chip. The derivatized chip surface was imaged with AFM, as reported in Fig. 3 panels a, b: the morphology of the surface was compatible with a homogeneous derivatization and showed a surface roughness (deepness) of about 15 nm. The z-axis value resulted smaller than what expected from the DLS, but in line with AFM and SEM data reported in Fig. 2. This could be explained by the softness and flexibility of the polymeric MIP NPs, that do shrink in the dry conditions used for the AFM imaging. The dips panel reported in Fig. 3c shows the changes in the reflection angle at the chip surface prior (black signal) and after (grey signal) the derivatization with MIP NPs. The dip shift at higher angles (shifts to the right: channel 1 from 65 to 65.3°, channel 2 from 65 to 65.4° and channel 3 from 65.1 to 65.5°) occurs when the mass on the sensing surface increases and could also be interpreted as a slight increment in hydrophobicity of the surface, once modified with the polymeric NPs [68].

### Sample injection time for the SPR measurements

**Fig. 4:**
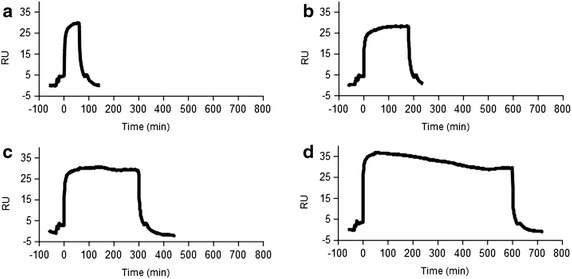
The injection time of Hepcidin-25 into the MIP NP SPR sensor. Different injection volumes of Hepcidin-25 were tested (from 30 to 300 µl) at a constant flow rate of 30 µl/min allowing the contact time between the ligand and the analyte to vary from 1 to 10 min (**a** 1 min, **b** 3 min, **c** 5 min, **d** 10 min). An injection volume of 90 µl (3 min) **b** was chosen for all the experiments

The SPR gives direct and real-time measurements of the binding events occurring at the sensor surface. As observed in Fig. 4, when the analyte is injected, the sensorgram shows three different phases: a steep increment in the association phase, a plateau when the equilibrium between association and dissociation is reached and the dissociation phase when the buffer flows. Here we aimed the determination of the level of bound analyte and to correlate it to the injected concentration. This is normally performed by measuring the binding at a fixed time of sample injection, once the equilibrium has been reached. Therefore, the optimal contact time between the analyte and the MIP NP surface was defined evaluating the sensor response to a solution at fixed concentration of Hepcidin-25 (140 pM) flowing on the MIP NP surface at a constant flow rate (30 µl/min) but by changing the injection volumes from 30 to 300 µl. This allowed the contact time between the analyte and the MIP NP surface to vary from 1 to 10 min. As indicated in Fig. 4, the equilibrium, represented by the plateauing of the signal, was obtained for an injection volume of 90 µl (3 min). Three minutes was the analysis time chosen for all the next experiments. The key advantage of the 3 min analysis is that the amount of sample required is fully compatible with future applications to real samples analysis, and is optimized respect to nanoMIP SPR using larger sample volumes (0.8–5 ml) [68, 69]. The rapid adsorption dynamic for the analyte was compatible with real time monitoring and can be related to the higher accessibility of the binding cavities in the nanomaterial [62, 70] and to its homogeneity, in comparison with macroMIPs and in accordance with other nanoMIPs [71–73].

### Study of the response of the MIP NP SPR sensor to Hepcidin-25

**Fig. 5:**
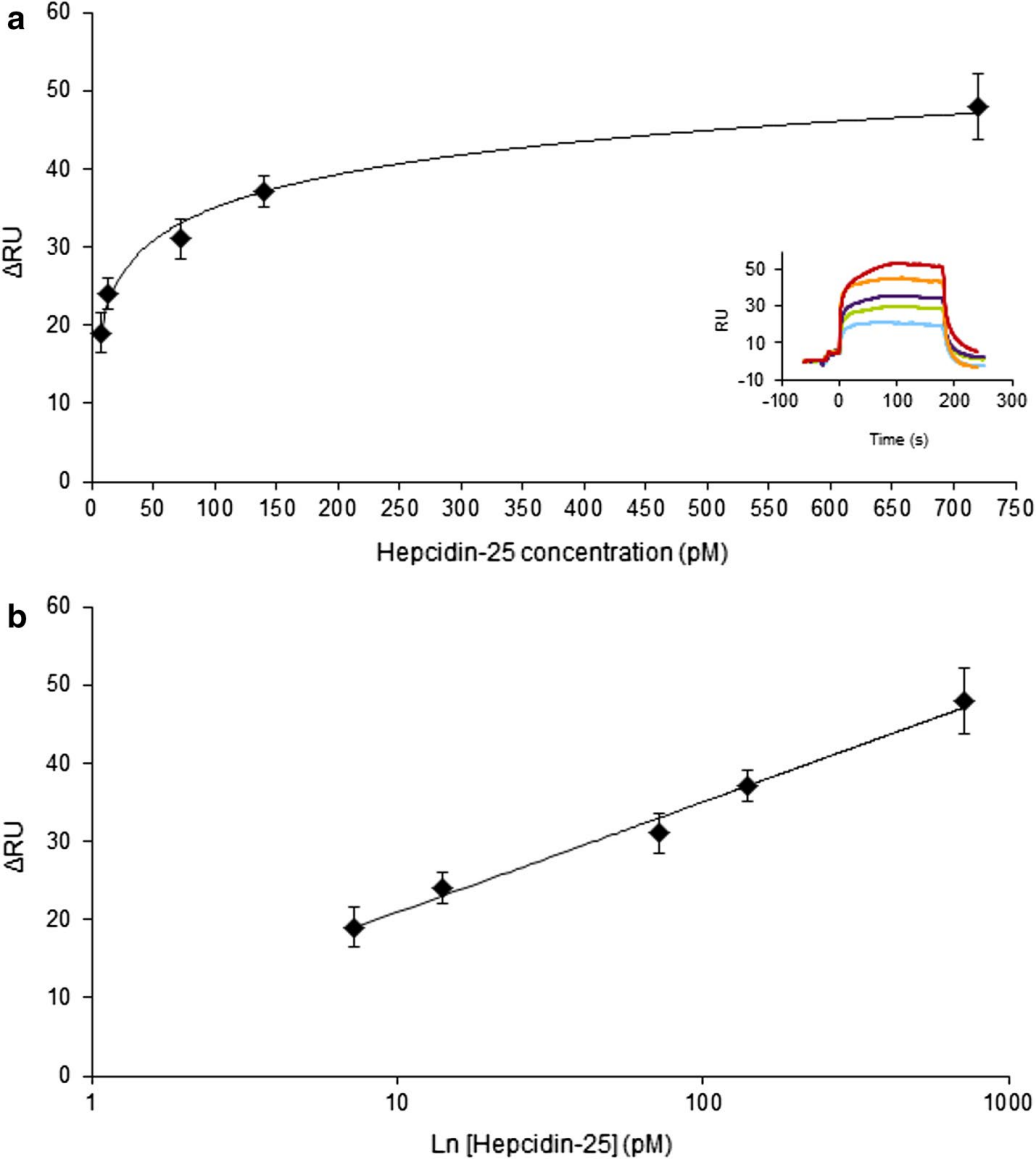
MIP NP SPR sensor response to Hepcidin-25. **a** Hepcidin-25 was injected at 7.2 pM (*light blue curve*), 14 pM (*green curve*), 72 pM (*violet curve*), 140 pM (*orange curve*) and 720 pM (*red curve*). The signal was recorded over timed and plotted as response units (RU). The sensorgrams of the MIP-NP-sensor response for Hepcidin-25 are shown in the *inset*. **b** Hepcidin-25 showed a log-linear response in the range 7.2–720 pM. A calibration curve was thus built for Hepcidin-25 by plotting the logarithm of the concentration vs ΔRU (Response Unit): the linear fit equation was f(x) = 6.07 ln(x) + 7.03 and the R^2^ was 0.98

The MIP NP SPR sensor response to Hepcidin-25 was studied. Hepcidin-25 was diluted in HBS and injected in a range of concentrations from picomolar to nanomolar to determine the sensitivity of the method. Hepcidin-25 produced a response in the range 7.2–720 pM as indicated in Fig. 5a, while higher concentrations gave a constant response due to the saturation of the sensor. Data of Fig. 5 are mediated out of five different set of measurements. The Limit of Detection (LOD) of the sensor for Hepcidin-25 was calculated as three times the standard deviation of the baseline reflectivity (see [73]) and resulted 5 pM.

The calibration curve for Hepcidin-25 was built by plotting the logarithm of the concentration vs. the Δ Response Units (ΔRU), as shown in Fig. 5b. The linear fit equation for the calibration curve was f(x) = 6.07 ln(x) + 7.03 and the R^2^ was 0.98.

The determination of Hepcidin-25 with the MIP NP SPR was at the picomolar level, with evident gain in sensitivity over an anti-Hepcidin-25 based SPR that was reported linear in the range 0.36–360 nM [74]. The low picomolar determination of Hepcidin-25 was reported so far with the sandwich immunoassays and in HPC-ICP-MS, respectively with LOD 35 and 140 pM [75] and in recent results with LC-HRMS where the reported LOD was 100 pM [47]. So far our MIP NP based sensor displayed the highest sensitivity for Hepcidin-25, while retaining the shortest analysis time.

### The selectivity of the MIP NP SPR sensor

**Fig. 6:**
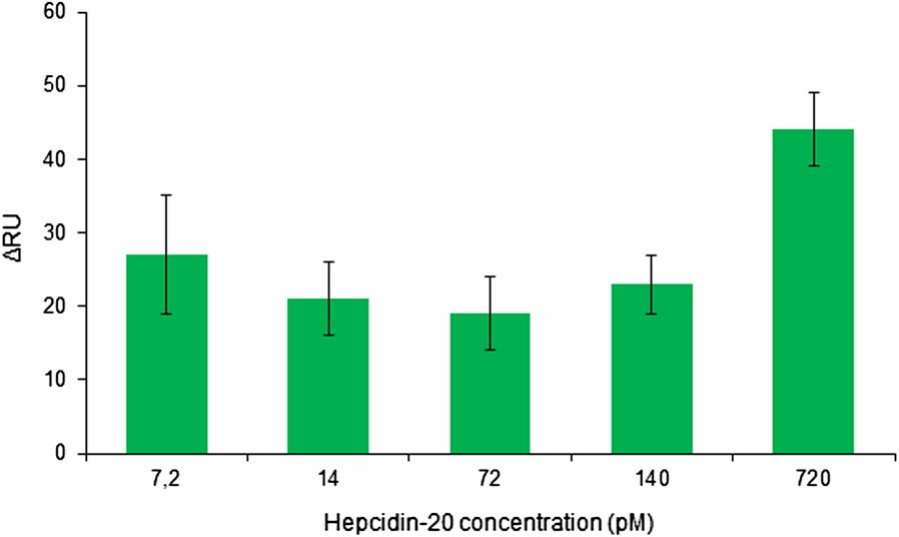
MIP NP SPR sensor response to Hepcidin-20. Hepcidin-20 was injected at increasing concentration in the sensor. The response to Hepcidin-20 was not proportional to the injected concentration, indicating the selectivity of the sensor

To evaluate the selectivity of the sensor the response to the truncated form of the hormone, Hepcidin-20, was studied. The results reported in Fig. 6 showed lack in proportionality between the increasing concentrations of Hepcidin-20 injected (7.2–720 pM) and the observed signal (ΔRU) of the sensor. This accounts for the selectivity of the sensor for Hepcidin-25 but not Hepcidin-20, indicating once more that the MIP NPs target specifically the N terminus of Hepcidin-25. However a significant increase in RU was observed for high loads of Hepcidin-20 (i.e. 720 pM). This could find explanation by the assumption that at concentrations of analyte close to the saturation limit of the sensing surface, unspecific interactions at the surface prevail. Such interactions, which cannot be previewed with the ITC experiments, warn us about the limits of operation of the MIP NP SPR sensor and indicate the necessity to reduce the unspecific binding prior to apply the sensor to the measurement of real samples.

### Stability and reproducibility of the MIP NP SPR

In order to ensure a constancy of the binding properties of the SPR surface, the sensor surface was regenerated prior to perform each set of analysis. This was obtained by a fresh immobilization of biotinylated-MIP NPs onto the chip surface, the level of regeneration was controlled by monitoring the change in the refractive angle. In these working conditions, the reproducibility of the sensor response, studied over five regeneration cycles, was performed by injecting a 7.2 pM solution of Hepcidin-25 and the calculated coefficient of variation was CV = 5.36 (corresponding to a CV % of 18 %).

### The sensor performance in serum samples

The analytical determination of Hepcidin-25 in the serum samples is the final purpose of our attempts to exploit frontier research in nanotechnology to the development of analytical methods. The MIP NP SPR sensor was put at test for the quantification of Hepcidin-25 directly in serum samples spiked with a final concentration of 14 or 50 pM of the hormone. It was recently reported about a SPR sensor based on a MIP nanosurface where the measurement of testosterone from serum samples was heavily hampered by the complexity of serum components, so that unspecific binding resulted in out of scale changes in the refractive index [73]. To avoid the same problem, we set up a protocol for the treatment of the serum samples, prior to the SPR analysis. Sera, spiked with Hepcidin-25 and diluted 250 times were filtrated on ultrafiltration membranes previously treated to reduce adsorption [76] (details in Additional file 5) and injected in the MIP NP SPR sensor. The Hepcidin-25 concentration in the serum samples was 14 and 50 pM. The measured contents in Hepcidin-25 were 15 ± 6 pM and 120 ± 67 pM. The measurements showed high standard deviations and scarce agreement between the known and measured concentrations, accounting for the unspecific adsorption of serum components at the chip surface. It can be hypothesized that the determination of Hepcidin-25 in serum samples shall need a calibration curve prepared in serum mimic. Alternatively the passivation of the chip surface after MIP NP derivatization shall be performed. Despite the performance of the MIP NP recognition element, to date, sera were still not quantifiable with our sensor.

## Conclusions

The present research demonstrated the possibility to produce polymeric NPs of 20–50 nm size, with high affinity and selectivity for Hepcidin-25 through the MIP technology. The MIP NPs were integrated into a SPR sensor and were proven to determine Hepcidin-25 in the picomolar range (7.2–720 pM) while discriminating the non regulative Hepcidin-20.

Our results stress the key role of MIP nanomaterials, in particular of the nano-sized MIP NPs, on the performance of the SPR sensing. Micro-MIP based SPR were reported to reach at best nanomolar sensitivities when particular polymeric properties were exploited, such as swelling, so to amply the shift in the reflectivity angle upon binding [77], or when the MIP polymers were admixed to conductive metallic nano-objects (Au NPs or Au nanostars), so to form discrete conductive composites to propagate the plasmonic wave [22, 78, 79]. However, the development of methods to growth controlled MIP nanofilms or NPs yielded to the achievement of SPR detection sensitivities in the picomolar range [69] and the formation of fractal superporous architectures in the nanoMIP allowed to reach the femtomolar level of detection (LOD 3.5 fM) [73]. Here we demonstrated the pM detection level gained by the use of fully polymeric MIP nanosized recognition elements in SPR. These significant sensitivities can be explained by: (1) the formation of high-affinity cavities in the material during the imprinting process and (2) the “almost” molecular dimensions of the MIP NPs allows these recognition elements to be placed in close proximity to the sensor surface and to offer easy access to the analyte.

The further key outcome of the present work is the useful indication for the progresses of the methodologies for the quantitative determination of Hepcidin-25. The MIP NPs demonstrated stability, high affinity and selectivity, thus the MIP NPs appear to be an ideal recognition element for Hepcidin-25 dosage. Integrated into the MIP NP SPR proved an effective mean for the fast detection of Hepcidin-25 (3 min) in a range of concentrations (picomolar) that is actually reached only with much more labour intensive and time-requiring approaches.

Moreover, the possibility offered by the MIP NP to lower down of about 1000 times the LOD for Hepcidin-25 detection, respect the actual measurements, can be of significant impact in medicine, allowing a clinical evaluation of the Hepcidin-25 levels in previously non measurable conditions.

On a broader perspective, the versatility of the imprinting process and the results here achieved suggest a widespread scenario of applications for the MIP NPs, such as their general employment as recognition element, or plastic antibodies, in analytical methods for a global targeting of actually undetected peptides and proteins that have key roles in biological processes and pathogenesis but for many reasons elude the existing dosages.

## Methods

Acrylamide (Aam), Methacrylic acid (MAA), *N*-t-butylacrylamide (TBAm), *N,N*′-methylenebisacrylamide (BIS), Diethylaminoethylacrylate (DEAEm), *N,N,N*′,*N*′-tetramethylethylenediamine (TEMED), ammonium persulfate (APS), sodium dodecyl sulfate (SDS), 4-(2-hydroxyethyl)-1-piperazineethanesulfonic acid (HEPES), sodium dihydrogen phosphate, sodium monohydrogen phosphate, sodium chloride, ethylendiaminetetraacetic acid (EDTA), Tween-20, acetic anhydride, dichloromethane (DCM), dimethylformamide (DMF), piperidin, di-tert-butyl dicarbonate, hydrazine, triisopropylsilane (TIS), dimethylsulphoxide (DMSO), *N,N*′-Diisopropylcarbodiimide (DIPEA), *O*-(2-Aminoethyl)-*O*′-[2-(biotinylamino)ethyl]octaethylene glycol (biotin-PEG amine), trifluoroacetic acid (TFA) were obtained from SIGMA-ALDRICH (Darmstadt, Germany). Amino acids (Fmoc and side chain protected), 1-Hydroxybenzotriazole (HOBt) *N,N,N*′,*N*′-Tetramethyl-*O*-(benzotriazol-1-yl)uronium tetrafluoroborate (TBTU), 4-Benzyloxybenzyl Alcohol Resin (Wang resin) was from StepBio (Bologna, Italy). Acetonitrile, methanol, ethanol were purchased from Vetrotecnica (Padova, Italy). Acryloxyethyl thiocarbamoyl Rhodamine B was from Polysciences, Inc. (Warrington, USA) BioCap sensor was purchased from ICx Technologies (Oklahoma City, USA), synthetic Hepcidin-25 and Hepcidin-20 were from Peptide International (Louisville, KY, USA). NR10 (NIDALGMEGR) was from TAG Copenhagen A/S (Copenhagen, Denmark). Serum samples were from healthy volunteer donors. PBS: 20 mM phosphate buffer pH 7.4, 150 mM NaCl, 0.01 % Tween. HBS: 10 mM Hepes pH 7.4, 0.15 M NaCl, 3.4 mM EDTA, 0.05 % Tween-20.

### Peptide synthesis

Peptide of sequence DTHFPI was obtained by solid phase peptide synthesis as C-terminal acids [80]. Nα-Fmoc-protected amino acids (threefold excess) were coupled by using TBTU (threefold excess) and DIPEA (4.5-fold excess). All amino acids were coupled twice. After Fmoc-deprotection with 25 % piperidine in DMF, the final cleavage of the peptide from the resin and the side chain protecting groups was performed with the mixture TFA/H2O/TIS 95/2.5/2.5. Peptides were purified by reverse-phase HPLC (RP-HPLC) and characterized by SELDI-MS.

### Synthesis of nanoparticles (NPs)

Acrylamide (Aam), Methacrylic acid (MAA) and *N*-t-butylacrylamide (TBAm) were added at 8, 8 and 4 % (w/v) respectively, *N*, *N*′-methylenebisacrylamide (BIS) was added at 80 % (w/v) and SDS was added at 0.02 % (w/v). Monomers and SDS were mixed in 10 ml of H_2_O to reach a total monomer concentration of 0.2 and 0.5 % (w/v) (14 and 35 mM, respectively). The solutions were filtered with a cut-off limit of 0.2 µm. The peptide DTHFPI was added to the MIP-vials at the final concentration of 32 and 200 µM. Vials were closed with rubber caps and were sonicated for 10 min. Afterwards, N_2_ was bubbled through the reaction mixture for 30 min. Following the addition of APS (0.04 % w/v) and TEMED (0.03 % w/v) the polymerization was carried out at 20 °C for 20 h. Nanoparticles (NPs) were suspended in 250 ml of 50 mM Tris and then extensively dialyzed against 3 l of pure water using a Vivaflow 50 system (100,000 MWCO) (Sartorius Stedim Italy, Firenze, Italy). Control, non-imprinted NPs (NIP) were synthesized using the same protocol but in absence of the template peptide. A second batch of NIP (NIP-R) was synthesized with the following composition: Aam (8 %), MAA (8 %), TBAm (2 %), Acryloxyethyl thiocarbamoyl Rhodamine B (2 %) and BIS (80 %). The yield of polymerization was calculated from the weight of the lyophilized NPs with respect to the total weight of the monomers added to the synthetic batch.

### Dynamic light scattering

Size distribution and polydispersity index (PDI) were determined by Dynamic Light Scattering (DLS) using a Zetasizer Nano ZEN3600 (Malvern Instruments Ltd, Worcestershire, UK) equipped with a 633 nm He–Ne laser. Particles were dissolved in PBS to a final concentration of 1 mg/ml and filtered 0.22 µm. A particle refractive index (RI) of 1590 and an absorption value of 0.01 were assumed and a detection angle of 173° was used.

### Static light scattering

The number average molar mass (Mn) was measured using 5 NP concentrations in the range 1–0.063 mg/ml after the instrument was calibrated with milliQ water. Raw data were used to build a Debye plot (KC/Rθ vs the particle concentration, where K is an optical constant, C is the particle concentration and Rθ is the sample Rayleigh ratio) whose linear fit intercept is 1/Mn. The molecular weight was estimated assuming a particle refractive index increment (dn/dC) of 0.17 ml/g and a spherical particle shape (Rg = 0.740 Rh) [81, 82]. The RI, viscosity, absorption values and the Rayleigh ratio were provided by the Zetasizer v.6.32 software (Malvern instruments Ltd, Worcestershire, UK) while the refractive index increment (dn/dC) was found in the American Polymer Standards Corporation.

### Zeta potential

The zeta potential was measured by dissolving NPs in 10 mM NaCl to a final concentration of 3 mg/ml and the measurements were performed using the universal dip cell (ZEN1002). The zeta potential was estimated by applying the Smoluchowski model (for small particles in aqueous media) and by using the water viscosity and RI values (RI = 1330 and viscosity = 0.8872 cP).

### AFM

For the AFM analysis NPs dissolved in water–ethanol solution at 1 mg/ml and diluted 10 and 100 times in isopropyl alcohol, then a drop of liquid was deposited onto clean silicon wafer and vacuum dried. The AFM images were acquired with a Unisolver P47 Scanning Probe Microscope from NT-MDT. Analyses were performed in semi-contact mode with a NSG10 silicon tip with a nominal radius of less than 10 nm (force constant is ~20 N/m and resonant frequency is ~300 kHz). Sample were initially scanned over an area of 10 × 10 µm^2^ in order to find a suitable place (clean from “dust” or big particles) for a more detailed analysis and then images were acquired with 1 × 1 µm^2^ scans or less.

### SEM

The secondary electron images of the NPs were obtained with a FE-SEM JEOL 7401, at 1–3 keV beam energies and 10 µA of emitted current.

For the SEM analyses NPs dissolved in water–ethanol solution at 1 mg/ml; the dispersion was further diluted 10 and 100 times in deionised MilliQ water; the dispersion was deposited onto a silicon wafer substrate and vacuum dried to evaporate the water. Part of the water crystallises around the particles as visible in the SEM micrographs.

The surface of mono-crystalline silicon wafer is smooth at near atomic level so it provides a good morphological contrast with the NPs. The contrast between the NPs and the silicon flat was enhanced by applying a 12° tilt of the sample holder. The nano-particles appear brighter than the flat substrate.

The low energy impinging electron beam did not cause any noticeable charging effect nor drift in the particles position.

### Isothermal titration calorimetry (ITC)

A Nano ITC Standard Volume (TA Instruments, Newcastle, USA) with a fixed gold cell was used to perform experiments. All the bindings were performed at physiological pH, in view of the final scope of the work which was to use the MIP NPs for the measurements of hepcidin-25 in serum samples. NPs (1.2 µM) and peptide (4 µM) were dissolved in PBS, sonicated for 10 min and then degassed under vacuum for 15 min prior to be loaded in the calorimeter. The reference cell was filled with 200 μl of degassed deionized water, the sample cell was filled with an equal volume of NPs (MIP/NIP) while 50 μl of peptide solution was loaded in the syringe. Each ITC experiment consisted of 12 injections of 4 μl at an interval of 300 s from each other with a stirring speed of 250 rpm. Experiments were performed at 25 °C. Data were fitted with independent sites model using the Nano Analyze Software v. 2.3.6 (TA Instruments, New Castle, DE, USA) and the dissociation constant (Kd), the reaction stoichiometry (n), the enthalpy, entropy and free energy variation (ΔH, ΔS and ΔG) were calculated. The equation selected was Independent site model equation: A = Mol Syringe, B = Mol Cell·n, C = −Ka A – Ka B − (Cell Volume/10^6^), Bound = (−C-sqr(C^2^ − 4Ka^2^AB))/(2Ka), Old Bound = Bound·(Total Cell Volume – Injection Volume)/Total Cell Volume, y = 10^9^·(Bound − Old Bound) dH.

### NP conjugation with biotin-PEG-amine

NPs (1 equivalent) and TBTU coupling reagent (*O*-(Benzotriazol-1-yl)-*N,N,N*′,*N*′-tetramethyluronium tetrafluoroborate) (1 equivalent) were dissolved in DMSO. Simultaneously biotin-PEG-amine (3 equivalents) was dissolved in DMSO as well and added to the reaction mixture. DIPEA (*N,N*-Diisopropylethylamine) (1.5 equivalents) was finally added and the pH of the solution was checked (DIPEA was added till the pH was in the range 8–9). The reaction lasted 1 h, then the mixture was dialyzed against abundant water.

### Characterization of biotinylated NPs

The size distribution of control and biotinylated NPs was evaluated by Dynamic Light Scattering (DLS). Control and biotinylated NIP-R, dissolved in water to 1 mg/ml, were filtered 0.22 µm and loaded onto a reverse-phase HPLC Ascentis^®^ C18 column (250 mm × 4.6 mm) (Sigma-Aldrich). The separation method was: flow rate 1 ml/min, injection loop 20 μl and detection wavelength 560 nm. NPs were eluted using the following gradient: 10 % B for 1 min, 10–90 % B in 20 min (A: water with 0.1 % trifluoroacetic acid (TFA) B: acetonitrile with 0.1 % TFA).

### Sensor chip derivatization

A BioCap chip (ICx Technologies, Oklahoma City, USA) was used: the BioCap surface is a carboxylated ethylene oxide surface with covalently immobilized NeutrAvidin™. Biotinylated MIPs (05 MIP32) were dissolved in HBS to a final concentration of 0.3 µM, sonicated for 10 min and centrifuged at 11,000 rpm for 5 min. The chip surface was derivatized with MIPs using an off-instrument protocol: 100 μl of supernatant were placed onto the chip surface and incubated for 2 h at room temperature under mild shaking. The chip surface was washed three times with 100 μl of HBS prior to be re-inserted in the instrument and was then let under flow (30 µl/min) until equilibration of the baseline.

### SPR measurements

Measurements were performed on a SensiQ Pioneer fully automated, three channel, surface plasmon resonance-based biosensing system (Icx Technologies, Oklahoma City, USA). The optical system is based on the SPREETA sensor developed by Texas Instruments, Inc. The sensor is designed in a Kretschmann [83] configuration where monochromatic light (870 nm) is reflected from the sensing surface over a range of incident angles thereby causing an angle-dependent reflectance minimum that is detected by the photodiode array. The SPR chip is a glass chip coated with a semitransparent 50 nm gold film, the optical reflection guide is composed of a glass prism with high quality optical surfaces that enable light from a light emitting diode (LED) to undergo SPR and to be reflected onto a photodiode array (PDA) on the same plane. Measurements were performed at a constant flow rate of 30 µl/min. Different injection volumes were tested (from 30 to 300 µl) allowing the contact time between the ligand and the analyte to vary from 1 to 10 min. An injection volume of 90 µl was chosen for all the experiments. Hepcidin-25 was diluted in HBS and injected at different picomolar and nanomolar to determine the linearity range. Afterwards Hepcidin-25 and Hepcidin-20 were compared in their linearity range.

### Serum samples analysis

Hepcidin-25-spiked serum samples and negative controls were filtered with 10 K molecular cut-off to eliminate high molecular weight contaminants and the flow-through was injected. 10 K cellulose centrifugal filter devices (Amicon^®^ Ultra-0.5, Millipore Corporation) were treated with the following protocol for the handling of highly hydrophobic proteins: filters were incubated overnight with 0.5 % Tween-20, rinsed with water, loaded with 400 µl of 100 mM glycine and centrifuged at 12,000 rpm for 10 min. Filters were incubated 2 h with 0.5 % Tween-20 again, loaded twice with 400 µl of deionized water and centrifuged. Serum samples were diluted 1:10 (v/v) with HBS and Hepcidin-25 was spiked to nanomolar concentrations. Samples were filtered with the previously treated devices (12,000 rpm for 10 min) and the flow-through was diluted 1:25 (v/v) with HBS again. The spiked Hepcidin-25 was therefore in the range 15–200 pM.

## Additional files

Additional file 1. Molecular modelling. Additional file 2. Synthesis of the NPs. Additional file 3. Isothermal titration calorimetry. Additional file 4. Characterization of biotinylated NPs. Additional file 5. Hepcidin filtration protocol for the handling of highly hydrophobic proteins.
