# Supplementary material for: Surface plasmon resonance based on molecularly imprinted nanoparticles for the picomolar detection of the iron regulating hormone Hepcidin-25
Source: J Nanobiotechnology. 2015 Aug 27;13:51. doi: 10.1186/s12951-015-0115-3 (PMC4549936; doi:10.1186/s12951-015-0115-3)
Supplement: Additional file 1. — Molecular modelling. [file 12951_2015_115_MOESM1_ESM.docx]

**Additional file 1: Molecular Modelling**

The structure of Hepcidin-25 peptide was downloaded from the PDB website (PDB: 2KEF). Diethylaminoethyl methacrylate (DEAEM), methacrylic acid (MAA) and acrylamide (Aam) were used as functional monomers. The 3D structures of these latter were downloaded from ZINC database [1]. The parameters for the monomers were derived using the PRODRG server [2].

The dockings were accomplished using a locally installed version of HADDOCK (High Ambiguity Driven biomolecular DOCKing) program [3,4] with default parameters. All calculations were performed with CNS1.2 [5]. Non-bonded interactions were calculated with the OPLS force field using a cutoff of 8.5 Å. The electrostatic potential was calculated by using a shift function, while a switching function (between 6.5 and 8.5 Å) was used to define the Van der Waals potential. The HADDOCK score is used to rank the generated poses. The N-terminus of Hepcidin-25, together with the monomers residues were considered as active restraints. In the first HADDOCK docking step, that is rigid body energy minimization, 1,000 structures were calculated. The 200 best solutions selected based on the intermolecular energy were used for the semi-flexible, simulated annealing followed by an explicit water refinement. Finally, the solutions were clustered using a cut-off of 7.0 Å root mean square deviation (RMSD) based on the pair-wise backbone RMSD matrix. For each docking experiment, the three most populated clusters were examined and the best structure for each of these three clusters was analyzed. The HADDOCK energy scores should be considered as a scoring function to predict the best complexes. Two docking tests were performed: 1. Hepcidin-25 with two molecules of DEAEM and two molecules of Aam ; 2. Hepcidin-25 with two molecules of MAA and two molecules of Aam.

**AD 1 Table 1.1.** Haddock score, final intermolecular energy and number of H bond interactions for Test 1.

| **Cluster#** | **HADDOCK score [a.u.] E_intermolecular_ [kcal/mol]** |  | **H bonds** |
| --- | --- | --- | --- |
| Cluster 1 | -59.2 -160.4 |  | 2 |
| Cluster 2 | -54.8 -57.2 |  | 2 |
| Cluster 3 | -47.1 -107.9 |  | 3 |

**AD 1 Table 1.2.** Haddock score, final intermolecular energy and number of H bond interactions for Test 2.

| **Cluster#** | **HADDOCK score [a.u.] E_intermolecular_ [kcal/mol]** |  | **H bonds** |
| --- | --- | --- | --- |
| Cluster 1 | -68.0 -243.7 |  | 8 |
| Cluster 2 | -72.0 -223.8 |  | 3 |
| Cluster 3 | -67.8 -275.4 |  | 7 |

1. Irwin JJ, Sterling T , Mysinger MM ,Bolstad ES, Coleman RG : **ZINC: A Free Tool to Discover Chemistry for Biology.**  J. Chem. Inf. Model 2012, 52:1757–1768.
2. Schüttelkopf W, van Aalten F. **"PRODRG: a tool for high-throughput crystallography of protein-ligand complexes"**. Acta Crystallogr 2004, D60:1355–1363.
3. Dominguez C, Boelens R, Bonvin AM: **HADDOCK: a protein-protein docking approach based on biochemical or biophysical information.** J Am Chem Soc 2003, 125:1731-1737.
4. de Vries SJ, van Dijk AD, Krzeminski M, van Dijk M, Thureau A, Hsu V, Wassenaar T, Bonvin AM: **HADDOCK versus HADDOCK: new features and performance of HADDOCK2.0 on the CAPRI targets.** Proteins 2007, 69:726-733.
5. Brunger AT, Adams PD, Clore GM, DeLano WL, Gros P, Grosse-Kunstleve RW, Jiang JS, Kuszewski J, Nilges M, Pannu NS, Read RJ, Rice LM, Simonson T, Warren GL. **Crystallography & NMR system: a new software suite for macromolecular structure determination**. Acta Crystallogr. D Biol. Crystallogr. 1998 , 54: 905–921.
