# Supplementary material for: Surface plasmon resonance based on molecularly imprinted nanoparticles for the picomolar detection of the iron regulating hormone Hepcidin-25
Source: J Nanobiotechnology. 2015 Aug 27;13:51. doi: 10.1186/s12951-015-0115-3 (PMC4549936; doi:10.1186/s12951-015-0115-3)
Supplement: Additional file 2. — Synthesis of the NPs. [file 12951_2015_115_MOESM2_ESM.docx]

**Additional file 2: Synthesis of the NPs**

**Additional file 2:** Table 2.1 reports the details of the recipes of the NPs synthesized.

**AD 2 Table 2.1:** NP composition.

| **NP batch** | **Template concentration** | | **Total monomer concentration** | | **Monomers**** | | | | | | **Cross-linker***** |
| --- | --- | --- | --- | --- | --- | --- | --- | --- | --- | --- | --- |
|  | **µM** | **µl*** | **%** | **mM** | **MAA** | | **Aam** | | **TBAm** | | **BIS** |
| 02 MIP32 | 32 | 53 | 0,2 | 14 | 49 µl | | 41 µl | | 37 µl | | 875 µl |
| 02 MIP200 | 200 | 333 | 0,2 | 14 | 49 µl | | 41 µl | | 37 µl | | 875 µl |
| 02 NIP | - | - | 0,2 | 14 | 49 µl | | 41 µl | | 37 µl | | 875 µl |
| 05 MIP32 | 32 | 53 | 0,5 | 35 | 122 µl | | 101 µl | | 90 µl | | 2190 µl |
| 05 MIP200 | 200 | 333 | 0,5 | 35 | 122 µl | | 101 µl | | 90 µl | | 2190 µl |
| 05 NIP | - | - | 0,5 | 35 | 122 µl | | 101 µl | | 90 µl | | 2190 µl |
|  | **µM** | **µl*** | **%** | **mM** | **MAA** | **Aam** | | **TBAm** | | **Rhoda** | **BIS** |
| NIP-R | - | - | 0,2 | 14 | 49 µl | 41 µl | | 19 µl | | 94 µl | 875 µl |

The reaction volume was 10 mL.

* DTHFPI peptide stock solution was 6 mM in water

** Monomer stock solutions were 2% (w/v)

*** BIS stock solution was 2% (w/v)
