# Supplementary material for: Surface plasmon resonance based on molecularly imprinted nanoparticles for the picomolar detection of the iron regulating hormone Hepcidin-25
Source: J Nanobiotechnology. 2015 Aug 27;13:51. doi: 10.1186/s12951-015-0115-3 (PMC4549936; doi:10.1186/s12951-015-0115-3)

# Additional file 3: Isothermal Titration Calorimetry (ITC)

The results of the ITC experiments are here below reported in AD 3 Figures 3.1. and AD 3 Figures 3.2.. MIP data were fitted with the independent site model.

**AD 3 Figure 3.1: ITC profile and binding curve of 05 MIP200 (panel A) and 05 NIP (panel B) titrated with DTHFPI (4 µM).**

**
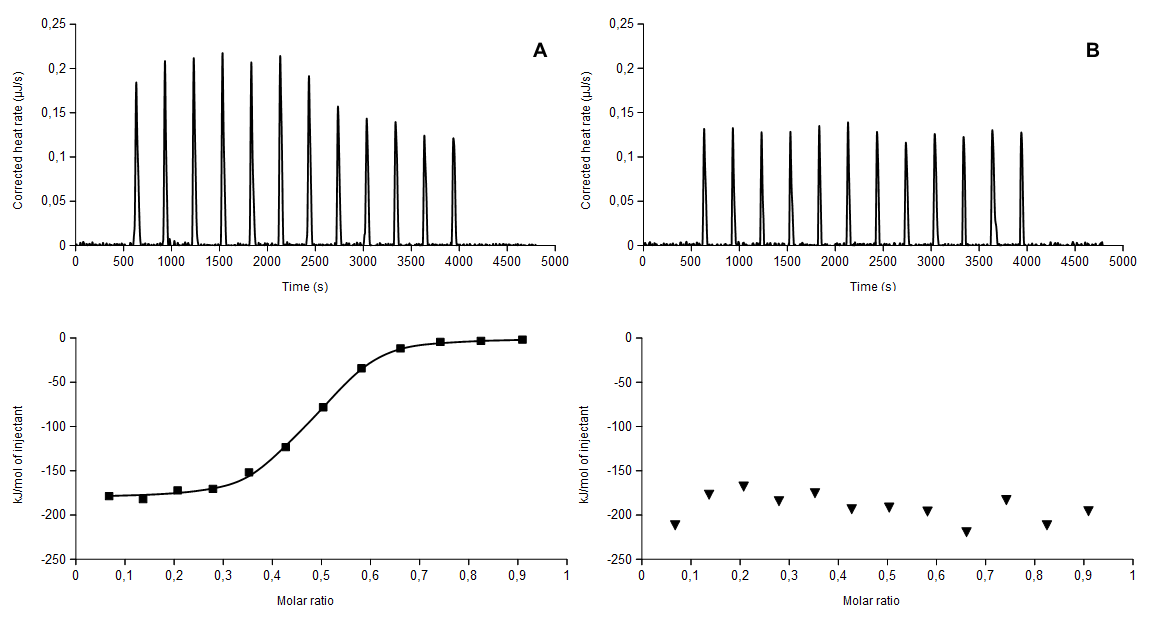
**

**AD 3 Figure 3.2: ITC profile and binding curve of 05 MIP200 titrated with Hepcidin-25 (4 µM) (panel A) and Hepcidin-20 (4 µM) (panel B).**


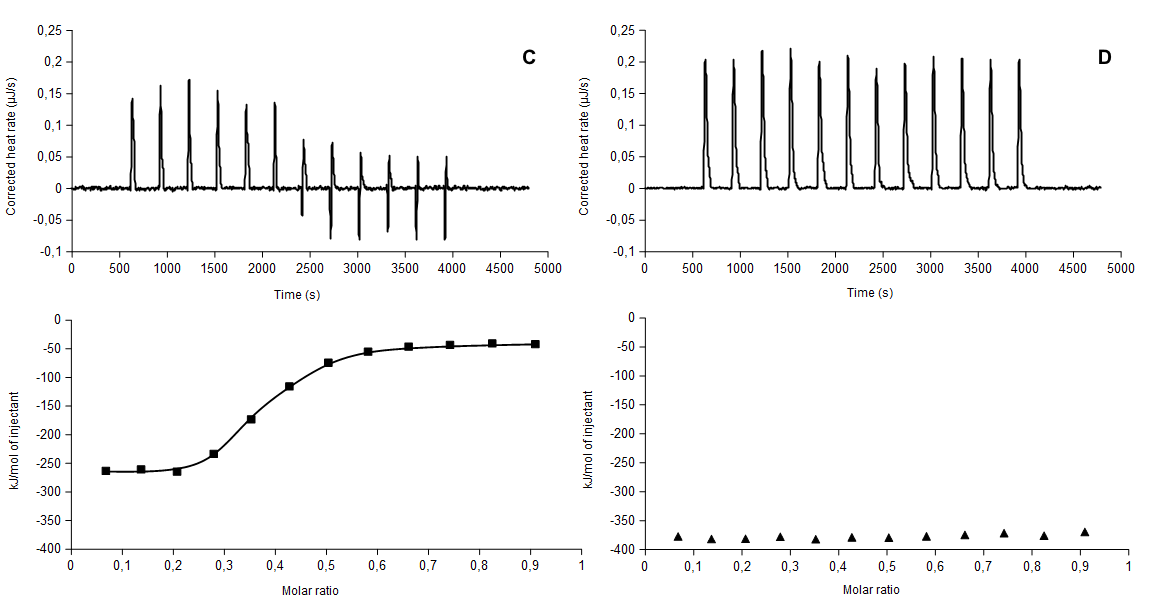

Supplement: Additional file 3. — Isothermal titration calorimetry. [file 12951_2015_115_MOESM3_ESM.docx]
