# Supplementary material for: Surface plasmon resonance based on molecularly imprinted nanoparticles for the picomolar detection of the iron regulating hormone Hepcidin-25
Source: J Nanobiotechnology. 2015 Aug 27;13:51. doi: 10.1186/s12951-015-0115-3 (PMC4549936; doi:10.1186/s12951-015-0115-3)

**Additional file 4: Characterization of biotinylated NPs**

The correct biotinylation was firstly assessed using NIP-R NPs, as the presence of the fluorescent monomer makes them easier to detect. After conjugation with biotin-PEG-amine, NIP-R NPs were loaded onto a HiTrap^TM^ Streptavidin HP column (GE Healthcare Bio-Sciences AB, Uppsala, Sweden) according to the manufacturer’s protocol. NPs were eluted with five volumes of Tris-HCl buffer with the addition of 6M urea. Absorption spectra of fractions collected from the column were recorded in the range 300-700 nm. The presence of NIP-R NPs (λ_max emission_ = 560 nm) in the first two fractions demonstrated the occurrence of biotinylation.

**AD 4 Figure 4.1: Spectrophotometric control of the biotinylated NPs.**

Absorption spectra (300-700 nm) of fractions collected from the Streptavidin column: fraction 1 (blue line), fraction 2 (red line), fraction 3 (light blue line), fraction 4 (green line).


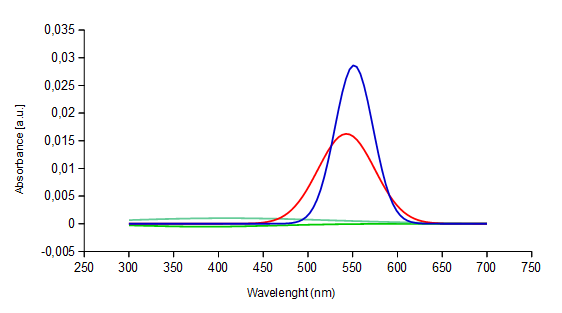


Size distribution of control and biotinylated NIP-R NPs was determined by Dynamic Light Scattering (DLS): control NPs showed a hydrodynamic diameter of 94 nm (± 7), an almost two-fold diameter was detected for biotinylated NP (172 nm ± 4). Similarly, control and biotinylated 05 MIP32 NPs were characterized in terms of size prior to be immobilized onto the SPR sensor chip: control 05 MIP32 showed a hydrodynamic diameter of 52 nm (± 2), a two-fold increment in size (139 nm ± 2) was measured for biotinylated 05 MIP32.

**AD 4 Figure 4.2: Size distribution of control and biotinylated NIP-R NPs.**


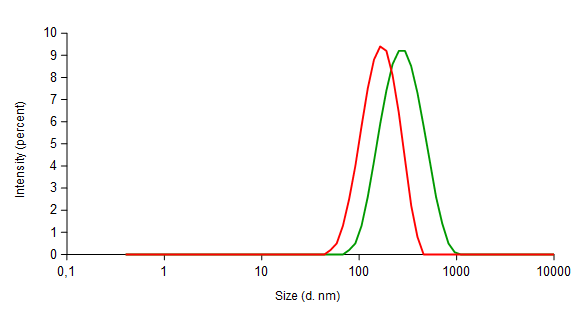

Supplement: Additional file 4. — Characterization of biotinylated NPs. [file 12951_2015_115_MOESM4_ESM.docx]
