# Supplementary material for: Surface plasmon resonance based on molecularly imprinted nanoparticles for the picomolar detection of the iron regulating hormone Hepcidin-25
Source: J Nanobiotechnology. 2015 Aug 27;13:51. doi: 10.1186/s12951-015-0115-3 (PMC4549936; doi:10.1186/s12951-015-0115-3)
Supplement: Additional file 5. — Hepcidin filtration protocol for the handling of highly hydrophobic proteins. [file 12951_2015_115_MOESM5_ESM.docx]

**Additional file 5: Hepcidin filtration protocol for the handling of highly hydrophobic proteins**

To avoid the issues reported in the direct SPR analysis of serum samples, sera were filtrated before the analysis. Nevertheless the tendency of Hepcidin-25 to stick to filters and plastic tube is evident [6] and it is not a negligible drawback.

We therefore evaluated different strategies for the filters treatment in order to limit the Hepcidin-25 depletion from samples after filtration.

To minimize the absorption effects due to both hydrophobic and electrostatic interactions, we treated the filters with a surfactant, i.e. tween 20, and a small charged molecules, as glycine. These two substances used in high concentrations should plentifully adsorb to filters/ plastic surfaces minimizing the further Hepcidin-25 sticking, as indicated also by the study of Lane et al. [7] The evaluation of the filter treatments was performed by absorption spectroscopy. Absorption spectra (200–290 nm) of Hepcidin-25 samples filtered onto pre-treated and not pre-treated cellulose filters (molecular weight cut-off 100K or 10K) were compared to the not filtered Hepcidin-25 sample (positive control) and are shown in Fig. 5.1 A and B.

**AD 5 Figure 5.1: Absorption spectra (200–290 nm) of for 6,5 µM Hepcidin-25 samples filtered on treated vs non treated filters.** Treated filter (red line), not treated filter (green line), 100K molecular weight cut-off (MWCO) (panel A) and 10K MWCO (panel B). 10K treated filters gave the best Hepcidin-25 recovery.


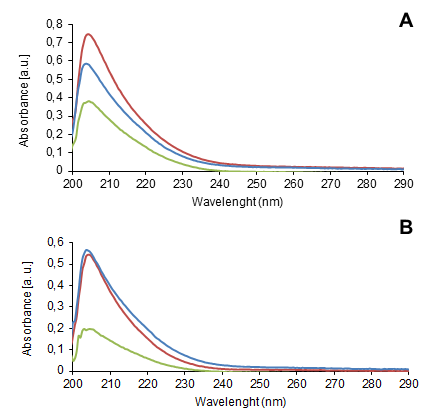


**AD 5 Figure 5.2:** **Efficiency of the filter pretreatment.** Graphical representation of the ratio between absorption (214 nm) of filtered/not filtered 6,5 µM Hepcidin-25 samples with pretreated (blue bars) and not pretreated (green bars) filters. The efficiency of this pre-treatment protocol for µM Hepcidin-25 filtration is evident.


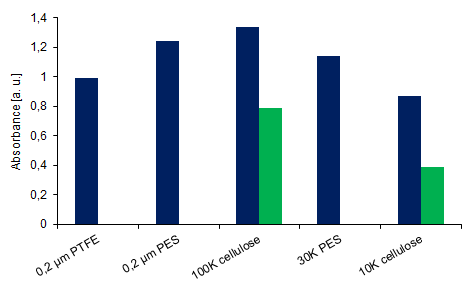


The protocol was assessed also for lower concentrations of Hepcidin-25. A SELDI-TOF MS analysis was performed using 10K cellulose filters and 20 nM Hepcidin-25 samples (Fig. 5.3). Quantitative results were obtained with the addition of the internal standard Hepcidin-24 to the filtered samples prior to MS analysis [8, 9]. SELDI-TOF MS data confirmed the validity of the protocol, in particular the treatment with both Tween-20 and glycine was selected, due to the better reproducibility of the results, when compared to the treatment with Tween-20 alone. Moreover the Hepcidin-24/Hepcidin-25 peak intensities ratio for the not filtered samples (0.63 ± 0.07) and for the filtered samples on device treated with Ttween-20 and glycine (0.68 ± 0.07) were coherent with literature data [10].

**AD 5 Figure 5.3: SELDI-TOF MS analysis of Hepcidin 25 after filtration on treated vs non treated filters.** Graphical representation of the ratio between Hepcidin-25/Hepcidin-24 peak intensities (Hepcidin-24 was added as internal standard). 20 nM Hepcidin samples and 10K cellulose filters were used. Filters treated with Tween and glycine, Tween alone and not treated were compared. A negative control (not filtered) was included. The Tween and glycine treatment was chosen since it is the most reproducible.


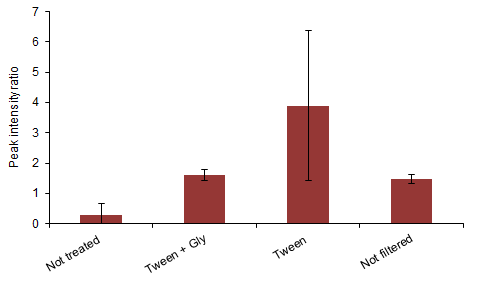


1. Castagna A, Campostrini N, Zaninotto F, Girelli D. **Hepcidin assay in serum by SELDI-TOF-MS and other approaches.** J Proteomics. 2010, 73:527-536Ra
2. Lane JS, Richens JL, Vere K-A,O’Shea P. **Rational targeting of subclasses of intermolecular interactions: elimination of nonspecific binding for analyte sensing**. Langmuir, 2014, 30:9457-9465.
3. Ward DG, Roberts K, Stonelake P, Goon P, Zampronio CG, Martin A, Jonhson PG, Iqbal T, Tselepis C. **SELDI-TOF-MS determination of hepcidin in clinical samples using stable isotope labelled hepcidin as an internal standard.** Proteome Sci. 2008, 6:28.
4. Swinkels DW, Girelli D, Laarakkers C, Joyce K, Campostrini N, Kemna EHJM, Tjalsma H. **Advances in Quantitative Hepcidin Measurements by Time-of-Flight Mass Spectrometry.** PLoS One. 2008, 3 :e2706. doi: 10.1371/journal.pone.0002706.
5. Kroot JJ, van Herwaarden AE, Tjalsma H, Jansen RT, Hendriks JC, Swinkels DW. **Second round robin for plasma hepcidin methods: first steps toward harmonization.** Am J Hematol. 2012, 87:977-83.
